# Polypharmacy and anticholinergic burden as risk factors for postoperative delirium in surgical medicine

**DOI:** 10.1007/s00391-024-02388-z

**Published:** 2025-01-06

**Authors:** Henriette Louise Moellmann, Soufian Boulghoudan, Julian Kuhlmann, Louisa Rahm, Helmut Frohnhofen

**Affiliations:** 1 Cranio-and-Maxillo Facial Surgery, University Hospital Düsseldorf, Moorenstraße 5, 40225 Düsseldorf, Germany; 2 Heinrich-Heine-Universität Düsseldorf, Universitätsstraße 1, 40225 Düsseldorf, Germany; 3 Orthopedics and Trauma Surgery, University Hospital Düsseldorf, Moorenstraße 5, 40225 Düsseldorf, Germany; 4 Faculty of Health, Department of Medicine, University Witten-Herdecke, Witten, Germany

**Keywords:** Postoperative delirium, Surgery, Polypharmacy, Anticholinergic burden, Geriatric assessment, Postoperatives Delir, Chirurgie, Polypharmazie, Anticholinerge Belastung, Geriatrisches Assessment

## Abstract

**Purpose:**

Polypharmacy is a widespread phenomenon in older patients. In particular, the anticholinergic burden of medication is an important risk factor for delirium due to age-related changes in the cholinergic system.

**Methods:**

Preoperative medication, including the calculation of the anticholinergic burden (ACB), was recorded in a prospective study (421 patients) to identify potential risks associated with medication intake. Postoperative delirium screening was carried out daily.

**Results:**

The study included 199 women (47.3%) and 222 men (52.7%) aged 80.8 ± 6.7 years and 78.8 ± 6.2 years, respectively. Antidepressants odds ratio (OR) 3.16 (95% confidence interval. CI, 1.51–6.64), antidiabetic drugs OR 2.53 (95% CI 1.27–5.03), neuroleptics OR 3.52 (95% CI 1.70–7.28) and Parkinson medication OR 5.88 (95% CI 1.95–17.7) showed a significantly higher risk for delirium. The ACB score revealed an anticholinergic burden in 43 patients (10.4%). The delirium rate was 25.6% (*n* = 11) and 11.0% (*n* = 40) had no anticholinergic burden. A significant correlation can be demonstrated with χ^2^(1) = 7.52, *p* = 0.006, Cramer’s V = 0.136. There was a 2.79-fold higher risk of delirium (OR 2.79, 95% CI 1.31–5.97).

**Conclusion:**

The standardized recording of medication is essential, especially when identifying patients at risk of suffering from delirium. The use of the ACB score to assess the anticholinergic burden is a simple and reliable screening tool and should be part of a preoperative geriatric assessment.

**Supplementary Information:**

The online version of this article (10.1007/s00391-024-02388-z) contains supplementary material, which is available to authorized users.

## Abstract (de)

**Hintergrund:**

Polypharmazie ist ein weit verbreitetes Phänomen bei älteren Patienten. Insbesondere die anticholinerge Belastung durch Medikamente ist ein wichtiger Risikofaktor für Delirien aufgrund altersbedingter Veränderungen im cholinergen System.

**Methoden:**

In einer prospektiven Studie (421 Patienten) werden die präoperative Medikation und die anticholinerge Belastung (ACB) erfasst, um mögliche Risiken im Zusammenhang mit der Medikamenteneinnahme zu identifizieren. Postoperativ wird täglich ein Delirscreening durchgeführt.

**Ergebnisse:**

An der Studie nahmen 199 Frauen (47,3 %; 80,8 ± 6,7 Jahre) und 222 Männer (52,7 %; 78,8 ± 6,2 Jahre) teil. Die Odds-Ratios (OR) für Antidepressiva (3,16; 95 % KI 1,51–6,64), Antidiabetika (2,53; 95 % KI 1,27–5,03), Neuroleptika (3,52; 95 % KI 1,70–7,28) und Parkinson-Medikamente (5,88; 95 % KI 1,95–17,7) zeigten ein signifikant höheres Risiko für ein Delir. Der ACB-Score ergab bei 43 Patienten (10,4 %) eine anticholinerge Belastung. Die Delirrate beträgt hier 25,6 % (*n* = 11), ohne anticholinerge Belastung 11,0 % (*n* = 40). Es lässt sich eine signifikante Korrelation (χ^2^(1)= 7,52; *p* = 0,006, Cramer’s V = 0,136) nachweisen. Es besteht ein 2,79-fach erhöhtes Risiko für ein Delir (OR 2,79; 95 % KI 1,31–5,97).

**Schlussfolgerung:**

Die standardisierte Erfassung der Medikation ist bei der Identifizierung von Patienten mit einem erhöhten Risiko für ein Delir von zentraler Bedeutung. Die Verwendung des ACB-Scores zur Beurteilung der anticholinergen Belastung sollte als einfaches und zuverlässiges Screening-Instrument in ein präoperatives geriatrisches Assessment implementiert werden.

## Introduction

The incidence and prevalence of postoperative delirium (POD) in older people are substantial, making the syndrome a significant healthcare challenge. The incidence of POD in older patients (depending on various factors, i.e. specific surgical procedure and preoperative condition) ranges from 10% to 50% [1]. Factors such as advanced age, comorbidities, severity of surgery, duration of anesthesia, perioperative complications and the use of anticholinergic medication can increase the risk of POD [2, 3]. Anticholinergic medication acts by blocking the effect of the neurotransmitter acetylcholine on cholinergic receptors, which leads to reduced activity of the cholinergic system and can cause various neurological symptoms in the brain, including various cognitive impairments and POD. Older people are exposed to increased sensitivity to the anticholinergic burden due to age-related changes in the cholinergic system [2, 4]. Polypharmacy in old age, defined as the simultaneous intake of five or more medications, is a widespread phenomenon that occurs more frequently with increasing age. The prevalence of polypharmacy in people over the age of 65 years in Germany is around 60% [5]. Polypharmacy in old age is associated with a variety of risks, including the risk of drug interactions, adverse drug reactions, medication misuse and an increased hospitalization rate. It also increases the risk of falls, hospitalization and mortality [6]. Regarding the risk of POD in old age, a major problem is the prescription of drugs with anticholinergic effects, which are associated with an increased risk of cognitive impairment, POD and other adverse effects [5, 6]. Optimizing medication according to the fit for the aged (FORTA) rules leads to a significant additional improvement in activities of daily living in older adults who are admitted to hospital for geriatric rehabilitation [7]. The aim of this study was to investigate the influence of the anticholinergic burden measured with the ACB score on the delirium rate.

## Material and methods

A comprehensive geriatric assessment was carried out in patients who underwent surgery under general anesthesia in the Department of Maxillofacial, Vascular, General and Trauma Surgery/Orthopedics during the data collection period (August 2021–October 2023). The patients’ state of health and individual risk factors were documented, these included age, gender, and body mass index (BMI) and the American Society of Anaesthesiologists (ASA) classification. Preoperative medication, including the calculation of the anticholinergic burden using the ACB score [8, 9], is recorded to identify potential risks associated with medication intake. The ACB scale classifies medications on a scale of 1–3, with higher values indicating a higher anticholinergic burden and a higher cognitive risk. The anticholinergic burden classification (ACB) was developed in response to the need to provide a standardized method for assessing the anticholinergic burden of medication. These are categorized according to their anticholinergic potential into 0 (no anticholinergic effects or negligible anticholinergic burden) to 3 (strong anticholinergic burden with pronounced anticholinergic effects) [10, 11]. Postoperatively, patients are examined daily regarding their general condition and the presence of delirium using the nursing delirium screening scale (NuDesc), the confusion assessment method (CAM) and the confusion assessment method in the intensive care unit (CAM-ICU).

### Statistical analysis

The values obtained from the measurements and the clinical data were analyzed using Jamovi version 1.6.9, (computer software, retrieved from https://www.jamovi.org, accessed on 19 March 2022, Sydney, Australia). A *p*-value of 0.05 was set for the hypothesis test [12, 13]. Bivariate relationships between relevant variables and the development of delirium (delirium vs. no delirium) were analyzed with χ^2^-tests. We estimated the odds ratios (OR) of the associations with 95% confidence intervals (CI). Statistical tests were two-sided, and significance was assessed at the alpha level of 0.05. A *p*-value of < 0.05 was defined as significant, a value of < 0.01 as very significant, and a value of < 0.001 as highly significant. Binomial logistic regression was used to determine the predicative power of the individual parameters.

## Results

**Fig. 1:**
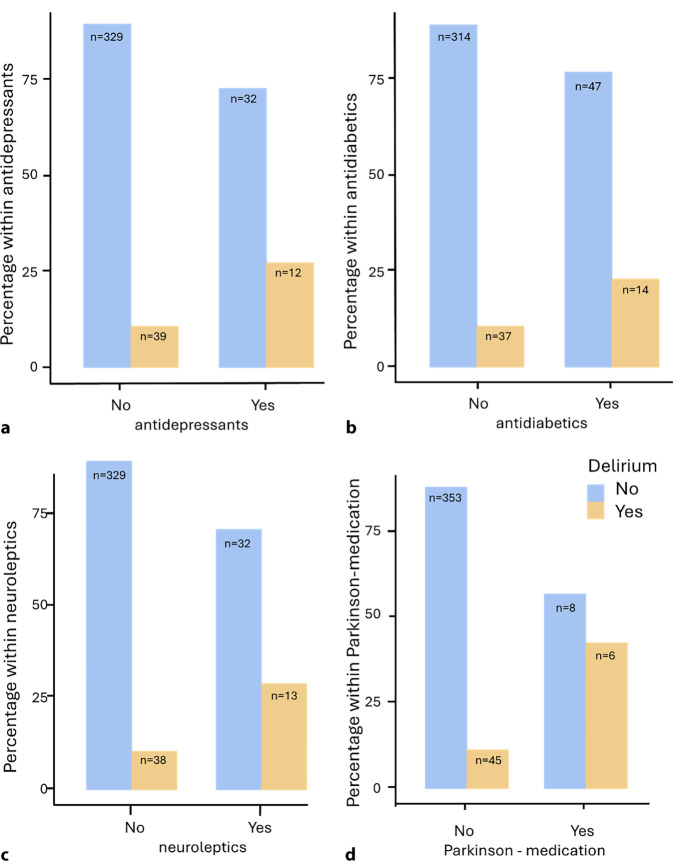
Illustration of delirium incidence depending on the administration of **a** antidepressants, **b** antidiabetics, **c** neuroleptics and **d** Parkinson medication

**Fig. 2:**
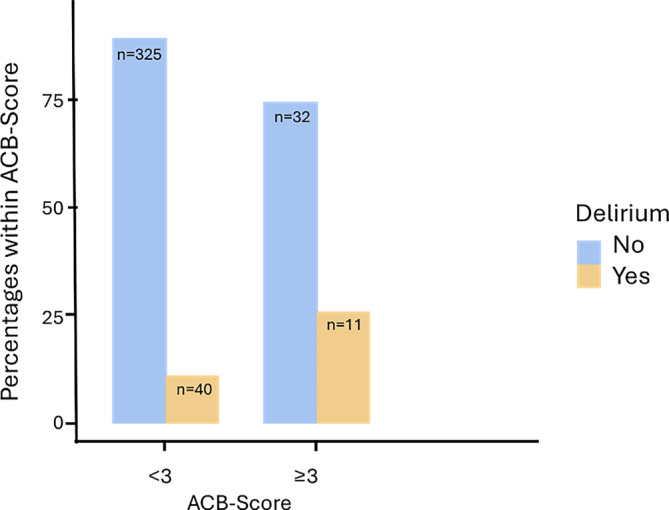
Illustration of the delirium rate in relation to an increased anticholinergic burden (ACB score ≥3)

The present collective consists of a total of 421 patients. Initial master data and parameters of the 199 women and 222 men (Table 1) and their correlation with the occurrence of delirium were analyzed (Table 2). The patients were taking 5.49–6.10 medications (interquartile range, IQR 5.00 medications). The difference in the number of medications in relation to the incidence of delirium was not significant with U = 8611, *p* = 0.491, r = 0.0584 at 5.10–6.67, IQR 2.00 (delirium) or 5.43–6.20, IQR (no delirium). Polypharmacy was present in 67.1% (*n* = 281) and it serves in this context as a surrogate marker for the multimorbidity of the patients. The delirium rate in patients with polypharmacy (*n* = 136) was 8.82% (*n* = 12), and 14.03% (*n* = 39) without polypharmacy (*n* = 278). A statistically significant correlation could not be demonstrated with χ^2^(1) = 3.52, *p* = 0.061, Cramer’s V = 0.135. The number of medications per group was considered (Supplementary Table 1). Individual medication groups show a significant correlation between delirium and the corresponding medication. The entire delirium rate was 12.3% (*n* = 51/416); however, due to the small size of the groups (*n* < 5) this can only be assessed to a limited extent. The following medication groups have an increased risk of delirium: antidepressants (OR 3.16, 95% CI: 1.51–6.64), antidiabetic drugs (OR 2.53, 95%CI: 1.27;5.03), neuroleptics (OR 3.52, 95%CI: 1.70;7.28) and Parkinson’s drugs (OR 5.88, 95%CI: 1.95;17.7; Fig. 1). A binomial logistic regression was performed to determine the effect of the medication to predict the likelihood of suffering from delirium. Of the 10 variables entered into the regression model, 2 significantly contributed in predicting delirium: antidiabetics (*p* = 0.026) and neuroleptics (*p* = 0.003), while the other variables showed no significant effect (Supplementary Table 2). They increase the likelihood of contracting a delirium. The ACB score (*n* = 413) is used to record the anticholinergic burden. The distribution of the scores in this collective is as follows (Supplementary Table 3). Therefore, 43 patients (10.4%) with 3 or more points have an increased anticholinergic burden and 370 patients do not have an increased anticholinergic burden. The delirium rate among patients with an increased anticholinergic burden is 25.6% (*n* = 11), among the other patients 11.0% (*n* = 40). A significant correlation can be demonstrated with χ^2^(1) = 7.52, *p* = 0.006, Cramer’s V = 0.136. There is a 2.79-fold higher risk of delirium (OR 2.79, 95%CI: 1.31;5.97; Fig. 2).

**Table 1:** Overview descriptives

Gender	Male	Female
*n* = 421	52.7% (*n* = 222)	47.3% (*n* = 199)
Age *n* = 419 (years)	78.8 ± 6.2	80.8 ± 6.7
Height	173.0 ± 9.25 cm	164 ± 13.7 cm
Body weight	78.5 ± 14.4 kg	68.0 ± 15.2 kg
BMI	25.7 ± 3.86	24.4 ± 5.46

*BMI* Body Mass Index

**Table 2:** Overview care level. Statutory care and ASA (American Society of Anesthesiologists) classification in relation to the delirium rate

Parameters	No.	Percentage (%)	Delirium rate
*Care level*	*n* *=* *310*	*–*	*–*
No/low	195	62.9	5.64% (*n* = 11)
Moderate	104	33.5	25.5% (*n* = 26)
High	11	3.5	36.4% (*n* = 4)
*Statutory care*			
Yes	30	9.6	36.7 (*n* = 11)
No	283	90.4	9.6% (*n* = 27)
*ASA (n* *=* *399)*			
I	17	4.3	9.1% (*n* = 1)
II	131	32.8	2.9% (*n* = 1)
III	227	56.9	14.3% (*n* = 6)
IV	24	6.0	No delirium

## Discussion

The incidence of POD in older patients is high and varies greatly depending on the study and type of surgery. In patients over 70 years of age, our study found an incidence of 12.3% in a patient population of 416 patients. Zhang et al. found an incidence of 17.1% in 637 hospitalized patients over 80 years old [14]. Wu et al. (2021) showed that several preoperative and postoperative risk factors are associated with an increased risk of POD in elective non-cardiac surgery under general anesthesia [15]. In addition to age, pre-existing cognitive diseases and comorbidities, polypharmacy is a significant predisposing factor. Our study showed a significant association between long-term medication with neuroleptics and the occurrence of POD according to the literature [16]. Campbell et al. (2020) investigated the incidence of POD in older patients taking antidepressants over the long term. Patients taking selective serotonin reuptake inhibitors (SSRI) or tricyclic antidepressants (TCA) had a significantly higher risk of developing POD [17]. Kronzer et al. (2020) investigated the effects of antidepressants on the risk of POD in older patients. The results showed that SSRIs and serotonin-norepinephrine reuptake inhibitors (SNRI) increased the risk of delirium, while the use of non-anticholinergic antidepressants had less impact on the risk of delirium. This emphasizes the importance of selecting appropriate antidepressants in older patients [18]. Johnson et al. investigated the long-term effect of levodopa on the occurrence of POD in Parkinson’s patients. Patients who took levodopa for a longer period of time had a higher risk of POD [19], long-term confusion and hallucinations [20]. Although they are generally well tolerated, they can significantly increase the risk of delirium in combination with other drugs administered perioperatively [21]. Opioids are frequently used analgesics, especially in older patients, and are also used in perioperative care [22]. Aldecoa et al. (2023) investigated the role of opioids in the development of POD [23]. Patients who received high doses of opioids perioperatively had a significantly higher risk of POD. They emphasized the need for cautious use of opioids and consideration of alternative pain management strategies to minimize the risk of delirium [24]. Zhang et al. (2023) compared postoperative patients in whom non-opioid-based or opioid-based pain management strategies were used. Patients treated primarily with opioids had a significantly higher incidence of POD compared to those receiving non-opioid analgesics [25]. Our study shows a significant correlation between the use of laxatives and the occurrence of POD. It should be mentioned that it is not the laxatives themselves but the underlying diseases that represent the risk factors. Constipation in older patients can be caused by a variety of factors such as reduced bowel motility, inadequate fluid intake and side effects of medications (opioids) [26]. Studies showed that preoperative constipation increases the incidence of POD in older patients [27, 28]. This emphasizes the need for a preventative approach to the treatment of constipation prior to surgical procedures but the laxative itself may increase the risk of POD via its side effects (e.g., dehydration, electrolyte disturbances). Kim et al. (2016) showed that the use of laxatives is associated with an increased risk of POD, especially when administered in high doses [29]. Whether the significant association is due to the drug side effects or the underlying disease cannot be accurately assessed retrospectively.

The relationship between the use of antidiabetic drugs and the occurrence of POD is multifaceted as some drug classes have been shown to have neuroprotective properties [30]. Particularly regarding polypharmacy, the various drug interactions can influence the risk of delirium. This may alter the pharmacokinetics and pharmacodynamics of antidiabetic drugs, which in turn may increase the risk of developing POD [31].

Mangla et al. (2018) analyzed data from older patients who underwent major abdominal surgery and showed that the use of diuretics such as furosemide was associated with an increased risk of developing POD due to electrolyte disturbances [32]. Neuman et al. (2017) investigated the association between preoperative diuretic use and PODs in older patients undergoing hip fracture surgery. Patients who had taken diuretics before surgery had a significantly higher risk of POD [33]. A meta-analysis by Leung et al. (2019) identified diuretics as a significant risk factor, particularly in older patients and those with pre-existing cognitive impairment [34].

## Conclusion

The systematic use of ACB-scores and similar assessment tools should be standardized in all healthcare facilities. These tools help to quantify the cumulative anticholinergic burden and enable a more targeted adjustment of medication. In addition, interdisciplinary collaboration between specialties such as geriatrics, neurology and anesthesiology can be crucial to minimize the risk of POD. Expanded training for healthcare professionals on risks of drugs with anticholinergic effects or other neurotoxic effects is essential. The steadily increasing polypharmacy of older patients therefore emphasizes the great importance and enormous potential of modifying the anticholinergic burden in the future to prevent or regulate the occurrence of postoperative delirium.

## Supplementary Information

Supplement Table 1: Correlation between delirium and medication

Supplementary Table 2: Overview model fit measures and coefficients: Models for predictability of the preoperative medication (polypharmacy, ACB score, antihypertensive medication, antidepressants, antidiabetics, neuroleptics, Parkinson medication, laxatives, opioid analgesics and non-opioid analgesics) of contracting a delirium. χ^2^(10) = 30.3, *p* < 0.001, Nagelkerke’s R2 = 0.136 *Note. The cut-off value is set to 0.5. Overall percentage of accuracy in classification was 87.1%, with a sensitivity of 3.9% and a specificity of 99.2%.

Supplementary Table 3: Anticholinergic burden in relation to the delirium rate

## Data Availability

The datasets used and/or analyzed during the current study are available from the corresponding author on reasonable request.
