# Supplementary material for: Polypharmacy and anticholinergic burden as risk factors for postoperative delirium in surgical medicine
Source: Z Gerontol Geriatr. 2025 Jan 6;58(3):203–8. doi: 10.1007/s00391-024-02388-z (PMC12048463; doi:10.1007/s00391-024-02388-z)
Supplement: Supplementary file 1 — Supplement Table 1: Correlation between delirium and medication [file 391_2024_2388_MOESM1_ESM.docx]

**Supplement Table 1:** Correlation between delirium and medication

|  | **Levels** | **Counts** | **% of Total** | **No Delirium** | **Delirium** | **Delirium incidence** |
| --- | --- | --- | --- | --- | --- | --- |
| Analgetics-Opioid | 0 | 373 | 89.4 % | 323 | 45 | 12.2% |
| n= 417; Χ^2^(2)=0.247, p= 0.0884, Cramers'V=0.0245 | 1 | 43 | 10.3 % | 37 | 6 | 14.0% |
|  | 2 | 1 | 0.2 % | 1 | 0 | 0.0% |
| Analgetics-Non-Opioid | 0 | 350 | 83.9 % | 302 | 43 | 12.5% |
| n= 417; Χ^2^(2)=0.148, p= 0.929, Cramers'V=0.0189 | 1 | 66 | 15.8 % | 58 | 8 | 7.6% |
|  | 2 | 1 | 0.2 % | 1 | 0 | 0.0% |
| Antibiotics | 0 | 409 | 98.1 % | 353 | 51 | 12.6% |
| n= 417; Χ^2^(1)=1.15, p= 0.283, Cramers'V=0.0529 | 1 | 8 | 1.9 % | 8 | 0 | 0.0% |
| Antihypertensive medication | 0 | 139 | 33.3 % | 119 | 19 | 13.8% |
| n= 417; Χ^2^(4)=10.4, p= 0.034, Cramers'V=0.159 | 1 | 169 | 40.5 % | 142 | 23 | 13.9% |
|  | 2 | 90 | 21.6 % | 84 | 6 | 6.7% |
|  | 3 | 18 | 4.3 % | 16 | 2 | 11.1% |
|  | 4 | 1 | 0.2 % | 0 | 1 | 100.0% |
| Sedatives, n=0 | 0 | 417 | 100.0 % | 361 | 51 | 12.2% |
| Glucocorticoids | 0 | 377 | 90.4 % | 324 | 49 | 13.1% |
| n= 417; Χ^2^(2)=2.17, p= 0.338, Cramers'V=0.0725 | 1 | 37 | 8.9 % | 34 | 2 | 5.6% |
|  | 2 | 3 | 0.7 % | 3 | 0 | 0.0% |
| Antidepressents | 0 | 371 | 89.0 % | 329 | 39 | 10.6% |
| n= 417; Χ^2^(3)=15.7, p= 0.001, Cramers'V=0.195 | 1 | 44 | 10.6 % | 31 | 11 | 26.2% |
|  | 2 | 1 | 0.2 % | 0 | 1 | 100.0% |
|  | 3 | 1 | 0.2 % | 1 | 0 | 0.0% |
| Antidiabetics | 0 | 355 | 85.1 % | 314 | 37 | 10.5% |
| n= 417; Χ^2^(4)=11.7, p= 0.020, Cramers'V=0.169 | 1 | 39 | 9.4 % | 27 | 11 | 28.9% |
|  | 2 | 18 | 4.3 % | 15 | 3 | 16.7% |
|  | 3 | 4 | 1.0 % | 4 | 0 | 0.0% |
|  | 4 | 1 | 0.2 % | 1 | 0 | 0.0% |
| Anticoagulants | 0 | 272 | 65.2 % | 233 | 36 | 13.4% |
| n= 417; Χ^2^(2)=0.823, p= 0.663, Cramers'V=0.0447 | 1 | 144 | 34.5 % | 127 | 15 | 10.6% |
|  | 2 | 1 | 0.2 % | 1 | 0 | 0.0% |
| Anticonvulsants | 0 | 383 | 91.8 % | 328 | 50 | 13.2% |
| n= 417; Χ^2^(3)=3.10, p= 0.376, Cramers'V=0.0868 | 1 | 28 | 6.7 % | 27 | 1 | 3.6% |
|  | 2 | 5 | 1.2 % | 5 | 0 | 0.0% |
|  | 3 | 1 | 0.2 % | 1 | 0 | 0.0% |
| Neuroleptics | 0 | 371 | 89.0 % | 329 | 38 | 10.4% |
| n= 417; Χ^2^(3)=14.1, p= 0.003, Cramers'V=0.185 | 1 | 39 | 9.4 % | 28 | 11 | 28.2% |
|  | 2 | 6 | 1.4 % | 3 | 2 | 40.0% |
|  | 3 | 1 | 0.2 % | 1 | 0 | 0.0% |
| Antirheumatics | 0 | 410 | 98.3 % | 355 | 50 | 12.3% |
| n= 417; Χ^2^(1)=0.0239, p= 0.877, Cramers'V=0.00761 | 1 | 7 | 1.7 % | 6 | 1 | 14.3% |
| Antihistaminics | 0 | 413 | 99.0 % | 358 | 50 | 12.3% |
| n= 417; Χ^2^(2)=1.36, p= 0.506, Cramers'V=0.0575 | 1 | 3 | 0.7 % | 2 | 1 | 33.3% |
|  | 2 | 1 | 0.2 % | 1 | 0 | 0.0% |
| Antiemetics | 0 | 415 | 99.5 % | 359 | 51 | 12.4% |
| n= 417; Χ^2^(1)=0.284, p= 0.5948, Cramers'V=0.0263 | 1 | 2 | 0.5 % | 2 | 0 | 0.0% |
| Bronchodilatators | 0 | 411 | 98.6 % | 355 | 51 | 12.6% |
| n= 417; Χ^2^(2)=0.860, p= 0.650, Cramers'V=0.0457 | 1 | 3 | 0.7 % | 3 | 0 | 0.0% |
|  | 2 | 3 | 0.7 % | 3 | 0 | 0.0% |
| Diuretics | 0 | 245 | 58.8 % | 217 | 26 | 10.7% |
| n= 417; Χ^2^(2)=2.79, p= 0.248, Cramers'V=0.0823 | 1 | 145 | 34.8 % | 121 | 23 | 16.0% |
|  | 2 | 27 | 6.5 % | 23 | 2 | 8.8% |
| Immunmodulators | 0 | 404 | 96.9 % | 349 | 51 | 12.8% |
| n= 417; Χ^2^(2)=1.75, p= 0.418, Cramers'V=0.0651 | 1 | 12 | 2.9 % | 11 | 0 | 0.0% |
|  | 2 | 1 | 0.2 % | 1 | 0 | 0.0% |
| Parkinson-Medication | 0 | 403 | 96.6 % | 353 | 45 | 11.3% |
| n= 417; Χ^2^(3)=16.6, p< 0.001, Cramers'V=0.201 | 1 | 9 | 2.2 % | 5 | 4 | 44.4% |
|  | 2 | 4 | 1.0% | 3 | 1 | 25.0% |
|  | 3 | 1 | 0.2% | 0 | 1 | 100.0% |
| Antiaaryhtmics | 0 | 216 | 51.8 % | 188 | 25 | 11.7% |
| n= 417; Χ^2^(2)=201, p= 0.905, Cramers'V=0.0221 | 1 | 192 | 46.0 % | 165 | 25 | 13.2% |
|  | 2 | 9 | 2.2 % | 8 | 1 | 11.1% |
| Sympathomimetics | 0 | 383 | 91.8 % | 328 | 51 | 13.5% |
| n= 417; Χ^2^(2)=5.07, p= 0.079, Cramers'V=0.111 | 1 | 28 | 6.7 % | 27 | 0 | 0.0% |
|  | 2 | 6 | 1.4 % | 6 | 0 | 0.0% |
| Parasympatholytics | 0 | 404 | 96.9 % | 350 | 49 | 12.3% |
| n= 417; Χ^2^(1)=0.112, p= 0.738, Cramers'V=0.0165 | 1 | 13 | 3.1 % | 11 | 2 | 15.4% |
| Muscle relaxants, n=0 | 0 | 417 | 100.0 % | 361 | 51 | 12.4% |
| Urologics | 0 | 372 | 89.2 % | 325 | 42 | 11.4% |
| n= 417; Χ^2^(1)=2.71, p= 0.100, Cramers'V=0.0810 | 1 | 37 | 8.9 % | 31 | 6 | 16.2% |
|  | 2 | 8 | 1.9 % | 5 | 3 | 37.5% |
| Antiplatelet drugs | 0 | 265 | 63.5 % | 224 | 36 | 13.8% |
| n= 417; Χ^2^(2)=1.53, p= 0.465, Cramers'V=0.0610 | 1 | 145 | 34.8 % | 131 | 14 | 9.7% |
|  | 2 | 7 | 1.7 % | 6 | 1 | 14.3% |
| Substitution - Vitamines/Trace elements/Electrolytes | 0 | 291 | 69.8 % | 256 | 32 | 11.1% |
| n= 417; Χ^2^(5)=4.38, p= 0.496, Cramers'V=0.103 | 1 | 66 | 15.8 % | 52 | 13 | 20.0% |
|  | 2 | 39 | 9.4 % | 35 | 4 | 10.3% |
|  | 3 | 18 | 4.3 % | 16 | 2 | 11.1% |
|  | 4 | 2 | 0.5 % | 1 | 0 | 0.0% |
|  | 5 | 1 | 0.2 % | 1 | 0 | 0.0% |
| Lipometabolism Medication | 0 | 223 | 53.5 % | 189 | 31 | 14.1% |
| n= 417; Χ^2^(2)=1.32, p= 0.518, Cramers'V=0.0565 | 1 | 171 | 41.0 % | 152 | 18 | 10.6% |
|  | 2 | 23 | 5.5 % | 20 | 2 | 9.1% |
| PPI | 0 | 254 | 60.9 % | 223 | 30 | 11.9% |
| n= 417; Χ^2^(1)=0.164, p= 0.685, Cramers'V=0.0200 | 1 | 163 | 39.1 % | 138 | 21 | 13.2% |
| Thyreostatics | 0 | 416 | 99.8 % | 360 | 51 | 12.4% |
| n= 417; Χ^2^(1)=0.142, p= 0.707, Cramers'V=0.0185 | 1 | 1 | 0.2 % | 1 | 0 | 0.0% |
| Bisphosphonates | 0 | 412 | 99.0 % | 356 | 51 | 12.5% |
| n= 416; Χ^2^(1)=0.572, p= 0.449, Cramers'V=0.0373 | 1 | 4 | 1.0 % | 4 | 0 | 0.0% |
| Phytopharmaceuticals | 0 | 409 | 98.1 % | 353 | 51 | 12.6% |
| n= 417; Χ^2^(2)=1.15, p= 0.283, Cramers'V=0.529 | 1 | 8 | 1.9 % | 8 | 0 | 0.0% |
| Gout medication | 0 | 378 | 90.6 % | 325 | 49 | 13.1% |
| n= 417; Χ^2^(2)=1.98, p= 0.372, Cramers'V=0.0693 | 1 | 38 | 9.1 % | 35 | 2 | 5.4% |
|  | 2 | 1 | 0.2 % | 1 | 0 | 0.0% |
| Endocrine Therapy | 0 | 337 | 80.8 % | 287 | 45 | 13.6% |
| n= 417; Χ^2^(1)=2.18, p= 0.140, Cramers'V=0.0727 | 1 | 80 | 19.2 % | 74 | 6 | 7.5% |
| AntineoplasticTherapy | 0 | 407 | 97.6 % | 351 | 51 | 12.7% |
| n= 417; Χ^2^(1)=1.45, p= 0.229, Cramers'V=0.0593 | 1 | 10 | 2.4 % | 10 | 0 | 0.0% |
| Laxantives | 0 | 383 | 92.1 % | 335 | 44 | 11.6% |
| n= 416; Χ^2^(3)=9.39, p= 0.024, Cramers'V=0.151 | 1 | 30 | 7.2 % | 23 | 6 | 20.7% |
|  | 2 | 2 | 0.5 % | 2 | 0 | 0.0% |
|  | 3 | 1 | 0.2 % | 0 | 1 | 100.0% |
