# Supplementary material for: Polypharmacy and anticholinergic burden as risk factors for postoperative delirium in surgical medicine
Source: Z Gerontol Geriatr. 2025 Jan 6;58(3):203–8. doi: 10.1007/s00391-024-02388-z (PMC12048463; doi:10.1007/s00391-024-02388-z)
Supplement: Supplementary file 2 — Supplementary Table 2: Overview model fit measures and coefficients: Models for predictability of the preoperative medication (polypharmacy, ACB score, antihypertensive medication, antidepressants, antidiabetics, neuroleptics, Parkinson medication, laxatives, opioid analgesics and non-opioid analgesics) of contracting a delirium. χ2(10) = 30.3, p < 0.001, Nagelkerke’s R2 = 0.136 *Note. The cut-off value is set to 0.5. Overall percentage of accuracy in classification was 87.1%, with a sensitivity of 3.9% and a specificity of 99.2%. [file 391_2024_2388_MOESM2_ESM.docx]

**Supplementary Table 2:** Overview Model Fit Measures and Coefficients: Models for predictability of the preoperative medication (polypharmacy, ACB-Score, antihypertensive medication, antidepressants, antidiabetics, neuroleptics, Parkinson-medication, laxatives, opioid analgesics and non-opioid analgesics) of contracting a delirium. χ²(10) = 30.3, p < 0.001, Nagelkerke’s R² = 0.136 *Note. The cut-off value is set to 0.5. Overall percentage of accuracy in classification was 87.1%, with a sensitivity of 3.9% and a specificity of 99.2%.

| Model Fit Measures | | | | | | | | | | | | | |  |  |  |  |
| --- | --- | --- | --- | --- | --- | --- | --- | --- | --- | --- | --- | --- | --- | --- | --- | --- | --- |
|  | | | | | | | | | **Overall Model Test** | | | | |  |  |  |  |
| **Model** | **Deviance** | | **AIC** | **BIC** | | | **R²_N_** | | **χ²** | | **df** | **p** | |  |  |  |  |
| 1 | 277 | | 299 | 343 | | | 0.136 | | 30.3 | | 10 | < .001 | |  |  |  |  |
| Model Coefficients - Delirium Yes/No | | | | | | | | | | | | | | | | | |
|  | | | | | **95% Confidence Interval** | | |  | | | | | | | | **95% Confidence Interval** | |
| **Predictor** | | **Estimate** | | | **Lower** | **Upper** | | **SE** | | **Z** | | | **p** | | **Odds ratio** | **Lower** | **Upper** |
| Intercept | | -2.4539 | | | -3.1296 | -1.778 | | 0.345 | | -7.1181 | | | < .001 | | 0.086 | 0.0437 | 0.169 |
| Polypharmacy >=5 | |  | | |  |  | |  | |  | | |  | |  |  |  |
| Yes - No | | 0.2504 | | | -0.6097 | 1.111 | | 0.439 | | 0.5707 | | | 0.568 | | 1.2846 | 0.5435 | 3.036 |
| ACB-score | |  | | |  |  | |  | |  | | |  | |  |  |  |
| Yes - No | | 0.3027 | | | -0.674 | 1.28 | | 0.498 | | 0.6075 | | | 0.544 | | 1.3536 | 0.5096 | 3.595 |
| Antihypertensive medication | |  | | |  |  | |  | |  | | |  | |  |  |  |
| Yes - No | | -0.3159 | | | -1.0787 | 0.447 | | 0.389 | | -0.8115 | | | 0.417 | | 0.7292 | 0.34 | 1.564 |
| Antidepressents | |  | | |  |  | |  | |  | | |  | |  |  |  |
| Yes - No | | 0.6329 | | | -0.3006 | 1.566 | | 0.476 | | 1.3288 | | | 0.184 | | 1.883 | 0.7404 | 4.789 |
| Antidiabetics | |  | | |  |  | |  | |  | | |  | |  |  |  |
| Yes - No | | 0.9032 | | | 0.108 | 1.698 | | 0.406 | | 2.2262 | | | 0.026 | | 2.4674 | 1.1141 | 5.465 |
| Neuroleptics | |  | | |  |  | |  | |  | | |  | |  |  |  |
| Yes - No | | 1.2009 | | | 0.4011 | 2.001 | | 0.408 | | 2.9431 | | | 0.003 | | 3.3231 | 1.4935 | 7.394 |
| Parkinson-medication | |  | | |  |  | |  | |  | | |  | |  |  |  |
| Yes - No | | 1.3362 | | | -0.0125 | 2.685 | | 0.688 | | 1.9418 | | | 0.052 | | 3.8044 | 0.9876 | 14.655 |
| Laxatives | |  | | |  |  | |  | |  | | |  | |  |  |  |
| Yes - No | | 0.3356 | | | -0.8178 | 1.489 | | 0.588 | | 0.5703 | | | 0.568 | | 1.3988 | 0.4414 | 4.433 |
| Opioid analgesics | |  | | |  |  | |  | |  | | |  | |  |  |  |
| Yes - No | | 0.0366 | | | -1.0371 | 1.11 | | 0.548 | | 0.0668 | | | 0.947 | | 1.0373 | 0.3545 | 3.035 |
| Non-opioid analgesics | |  | | |  |  | |  | |  | | |  | |  |  |  |
| Yes - No | | -0.4402 | | | -1.4154 | 0.535 | | 0.498 | | -0.8848 | | | 0.376 | | 0.6439 | 0.2428 | 1.707 |
| *Note. Estimates represent the log odds of "Delirium Yes/No= Yes" vs. "Delirium Yes/No = No", With a coefficient of determination of R² = 0.136, a sample size of 421 and a significance level of α = 0.05, the statistical power of 6 predictors would be 1-β = 1. The statistical power indicates the probability of committing an error of the 2nd kind. Here, the probability of committing a 2nd type of error would be 0%. In 0% of cases, the test would not indicate significance, even if it were actually significant [22].* | | | | | | | | | | | | | | | | | |
