# Supplementary material for: Polypharmacy and anticholinergic burden as risk factors for postoperative delirium in surgical medicine
Source: Z Gerontol Geriatr. 2025 Jan 6;58(3):203–8. doi: 10.1007/s00391-024-02388-z (PMC12048463; doi:10.1007/s00391-024-02388-z)
Supplement: Supplementary file 3 — Supplementary Table 3: Anticholinergic burden in relation to the delirium rate [file 391_2024_2388_MOESM3_ESM.docx]

**Supplementary Table 3: Anticholinergic burden in relation to the delirium rate**

| Frequencies of ACB-score | | | | | | | |
| --- | --- | --- | --- | --- | --- | --- | --- |
| **Levels** | | **Counts** | | **% of Total** | **Cumulative %** | | |
| 0 |  | 212 |  | 51.3 % |  | 51.3 % |  |
| 1 |  | 108 |  | 26.2 % |  | 77.5 % |  |
| 2 |  | 50 |  | 12.1 % |  | 89.6 % |  |
| 3 |  | 22 |  | 5.3 % |  | 94.9 % |  |
| 4 |  | 8 |  | 1.9 % |  | 96.9 % |  |
| 5 |  | 6 |  | 1.5 % |  | 98.3 % |  |
| 6 |  | 4 |  | 1.0 % |  | 99.3 % |  |
| 7 |  | 2 |  | 0.5 % |  | 99.8 % |  |
| 8 |  | 1 |  | 0.2 % |  | 100.0 % |  |
